# Mechanism of Stability Enhancement for Adiponitrile High Voltage Electrolyte System Referring to Addition of Fluoroethylene Carbonate

**DOI:** 10.3389/fchem.2020.588389

**Published:** 2020-10-06

**Authors:** Zhao Fang, Zekun Zheng, Wudan Cheng, Xingliang Zhang, Kenan Zhong, Linbo Li

**Affiliations:** ^1^School of Metallurgical Engineering, Xi'an University of Architecture and Technology, Xi'an, China; ^2^Shaanxi Province Metallurgical Engineering and Technology Research Centre, Xi'an, China

**Keywords:** lithium ion battery, adiponitrile, fluoroethylene carbonate, additives, γ-butyrolactone, high voltage

## Abstract

In order to improve the stability of high voltage electrolyte for 5 V-level LiNi_0.5_Mn_1.5_O_4_ cathode material, adiponitrile (ADN) with high oxidation stability was selected as the main solvent, meanwhile, 2% fluoroethylene carbonate (FEC) as the additive with good film forming effect was also used. And then, the effect of 2 mol L^−1^ LiBF_4_-GBL/ADN+2% FEC on the electrochemical performance of LiNi_0.5_Mn_1.5_O_4_ was explored at room temperature. The electrolyte system containing FEC can improve the cycle stability of the battery. At 1 C rate, the cycle capacity retention rate can reach 83% after 100 cycles, while the capacity retention rate of the electrolyte system without FEC and the ordinary commercial electrolyte system is only 77 and 68%, respectively. Besides, the rate performance of the battery with the addition of FEC also shows excellent performance, however, this kind of advantage is not obvious under the conditon of large rate. In addition, under the conditon of the synergistic effect between adiponitrile and fluoroethylene carbonate, the high-voltage electrolyte exhibits the good compatibility and lithium reversibility in the full cell with Li_4_Ti_5_O_12_ as the negative electrode.

## Introduction

Improving the energy density of the battery can be carried out from the perspective of increasing the operating voltage of the battery. It is necessary to explore a high-voltage resistant electrolyte which matches the 5 V-level high-voltage cathode material LiNi_0.5_Mn_1.5_O_4_. At present, researchers have invested a lot of efforts in the research of 5 V high-voltage cathode materials. LiNi_0.5_Mn_1.5_O_4_ is a new material obtained by modifying the structure and element reference of the original spinel-type material LiMn_2_O_4_ as well as adding a small amount of nickel element, and its specific capacity is the same as that of lithium manganite. While, the working voltage is 17% higher than that of lithium manganese high-pressure materials (Talyosef et al., 2007). The positive electrode spinel type material LiNi_0.5_Mn_1.5_O_4_ charge and discharge platform up to 4.7 V (Zhou et al., 2012), it's specific capacity is 146.7 mAh g^−1^, the actual specific capacity is 125~135 mAh g^−1^, the reversible specific capacity can reach 133 mAh g^−1^ and the energy density is up to about 650 Wh kg^−1^ (Kim et al., 2004; Santhanam and Rambabu, 2010; Zhou et al., 2012; Wang, 2015). The high operating voltage platform meets the demand for high energy density of lithium ion batteries. However, the main problem of LiNi_0.5_Mn_1.5_O_4_ is that during cycling, manganese ions dissolve from the overall material into the electrolyte, especially at higher temperatures and pressures, resulting in a rapid decline in battery discharge specific capacity and columbic efficiency will also fall (Yoon et al., 2012; Manthiram et al., 2014). Also under high voltage conditions, LNMO-based batteries containing organic carbonate solvent electrolytes have serious capacity decay problems, which are related to electrolyte decomposition and simultaneous degradation reactions at the electrode/electrolyte interface, especially at high temperatures. Due to the thermal instability of LiPF_6_/carbonate electrolyte, the evolution of toxic decomposition products, and the accelerated decomposition reaction between the electrolyte and the electrode, the dissolution of manganese ions and nickel ions increases, the irreversible capacity of the battery increases, and the electrochemical cycle performance is seriously degraded (Arunkumar and Manthiram, 2005). Xiang et al. (2008) added dimethyl methylphosphonate (DMMP) flame retardant additives to 1M LiPF_6_/EC+DMC, although the safety performance of the LiNi_0.5_Mn_1.5_O_4_-Li_4_Ti_5_O_12_ full battery was improved, but the overall working voltage did not meet the demand for high energy density.

According to research, fluorine atoms are extremely electronegative and have strong electron-withdrawing capabilities. The fluorinated solvent of highest occupied orbital energy level and the lowest unoccupied orbital energy level are lower than the traditional carbonate solvent, that is, the oxidation potential and reduction potential of the fluorinated solvent are higher than the traditional carbonate solvent (Michan et al., 2016). Fluorinated ethylene carbonate (FEC) is often used as a high-pressure resistant solvent or negative electrode solid electrolyte interface film (SEI film) film-forming add itive, and its film-forming effect is good (Choi et al., 2006; Wu et al., 2013; Markevich et al., 2014). Although the literature shows TMSP additives also help MCMB anode and LiNi_0.5_Mn_1.5_O_4_ cathode to form a thicker and more conductive SEI layer, for Li_4_Ti_5_O_12_ anode, FEC can exert better compatibility (Lu et al., 2019). In the current work, high concentration LiBF_4_/GBL electrolyte solution is used to improve the charge / discharge performance of graphite anode and LiNi_0.5_Mn_1.5_O_4_ cathode (Sawai and Ohzuku, 2003; Ufheil et al., 2005). In previous studies, researchers usually used γ-butyrolactone (GBL) or glutaronitrile (GLN) as a co-solvent containing more than 10% in the standard electrolyte system, which improved the cycle performance of the battery (Duncan et al., 2013). Adiponitrile (ADN) shows the best thermal stability, low viscosity and high dielectric constant in many dinitrile-based solvents (Abu-Lebdeh and Davidson, 2009; Isken et al., 2011; Douaa et al., 2018). The most remarkable feature of this kind of substance is that it can still ensure that the electrolyte has an electrochemical window of about 7.0 V when used alone, and can still maintain 6.0~6.5 V in binary or ternary electrolyte (Kirshnamoorthy et al., 2018). Adiponitrile (ADN) has high oxidation stability under high pressure. According to reports, using ADN and DMC as binary solvents and adding fluoroethylene carbonate can improve the cycle performance of graphite/LFMP full batteries at 4.4 V, and FEC makes the battery cycle efficiency reach 99.9% or more (Ehteshami et al., 2018).

Based on the demand for high-energy density and high-power lithium-ion batteries and the problems and the analysis of the high-pressure resistant electrolyte mentioned above, this paper is committed to optimizing the electrochemical performance of electrolyte for the high-voltage material LiNi_0.5_Mn_1.5_O_4_. The binary solvent system [LiBF_4_/γ-butyrolactone (GBL) and adiponitrile (ADN)] with a wide electrochemical window was used to obtain an electrolyte solution with high oxidation stability. And then, fluoroethylene carbonate (FEC) with film-forming as an additive was used to increase the overall oxidation stability of the electrolyte and reduce the interface resistance, finally optimize the overall performance of the high-pressure electrolyte. Meanwhile, the mechanism of synergistic effect between ADN and FEC on high voltage system was also analyzed.

## Experimental Section

### Electrolyte Preparations

The electrolyte is prepared in argon-filled atmosphere, and the moisture and oxygen values in the glove box are required to be <2 ppm. Four electrolytes need to be prepared, the reference electrolyte: commercial electrolyte 1.0 M LiPF_6_ (EC:EMC = 3:7, volume ratio); comparison electrolyte 1st: 2 vt.% FEC additive was added to 1.0 M LiPF_6_ (EC:EMC = 3:7, volume ratio) standard electrolyte; comparison electrolyte 2nd: 2 mol L^−1^ LiBF_4_-GBL/ADN (1:1, volume ratio); target electrolyte: 2 mol L^−1^ LiBF_4_-GBL/ADN (1:1, volume ratio)+2% FEC.

### Cell Preparations

LiNi_0.5_Mn_1.5_O_4_ active material, Super P and polyvinylidene fluoride (PVDF) according to the mass ratio of 93:3:4 (Li_4_Ti_5_O_12_ is 86:7.7:6.3) are mixed, and apply the prepared slurry to a clean aluminum foil with a spatula. The coating thickness is controlled to 0.1 mm. Next, the coated pole pieces are dried in a blast drying oven for half an hour, and then taken out and punched into 15 mm pole pieces with a punching machine. The cut pole pieces are moved to a 120°C vacuum drying box for 12 h for drying to remove NMP and moisture.

### Measurements

Assemble the batteries of LiNi_0.5_Mn_1.5_O_4_/Li and LiNi_0.5_Mn_1.5_O_4_ with case model CR2032. The electrode is a lithium sheet with a diameter of 15.6 mm, and the separator is a polypropylene film with a diameter of 20 mm. After completion, it is necessary to stand at a constant temperature for 12 h to fully contact the separator and the pole piece for the electrolyte. Finally the batteries are subjected to a constant current charge and discharge test: LiNi_0.5_Mn_1.5_O_4_/Li half-cells are pre-circulated with a current of 0.1 C for 3 turns to achieve the formation effect, so that the active material can fully exert its performance, and then cycled with a current of 1 C for 100 turns, with a voltage range of 3~4.9 V. The voltage range of LiNi_0.5_Mn_1.5_O_4_/Li_4_Ti_5_O_12_ full battery is 2–3.5 V, test the cycle and rate performance of the batteries. Cyclic voltammetry (CV) curve scanning uses LiNi_0.5_Mn_1.5_O_4_ electrode as working electrode, metal lithium sheet as reference electrode and counter electrode, the scan voltage range is 2.7–4.5 and 3–4.9 V with a scan voltage rate of 0.1 mV s^−1^. The frequency range of electrochemical AC impedance is 0.01–105 Hz, and the disturbance amplitude is 5 mV. Scanning electron microscope (Zeiss supra55), X-ray photoenergy spectroscopy (Japan Rigaku Smartlab9) and X-ray diffraction (Escalab 250Xi) were used to characterize and analyze the mechanism of action of electrolytes and materials.

## Results and Discussion

**Figure 1:**
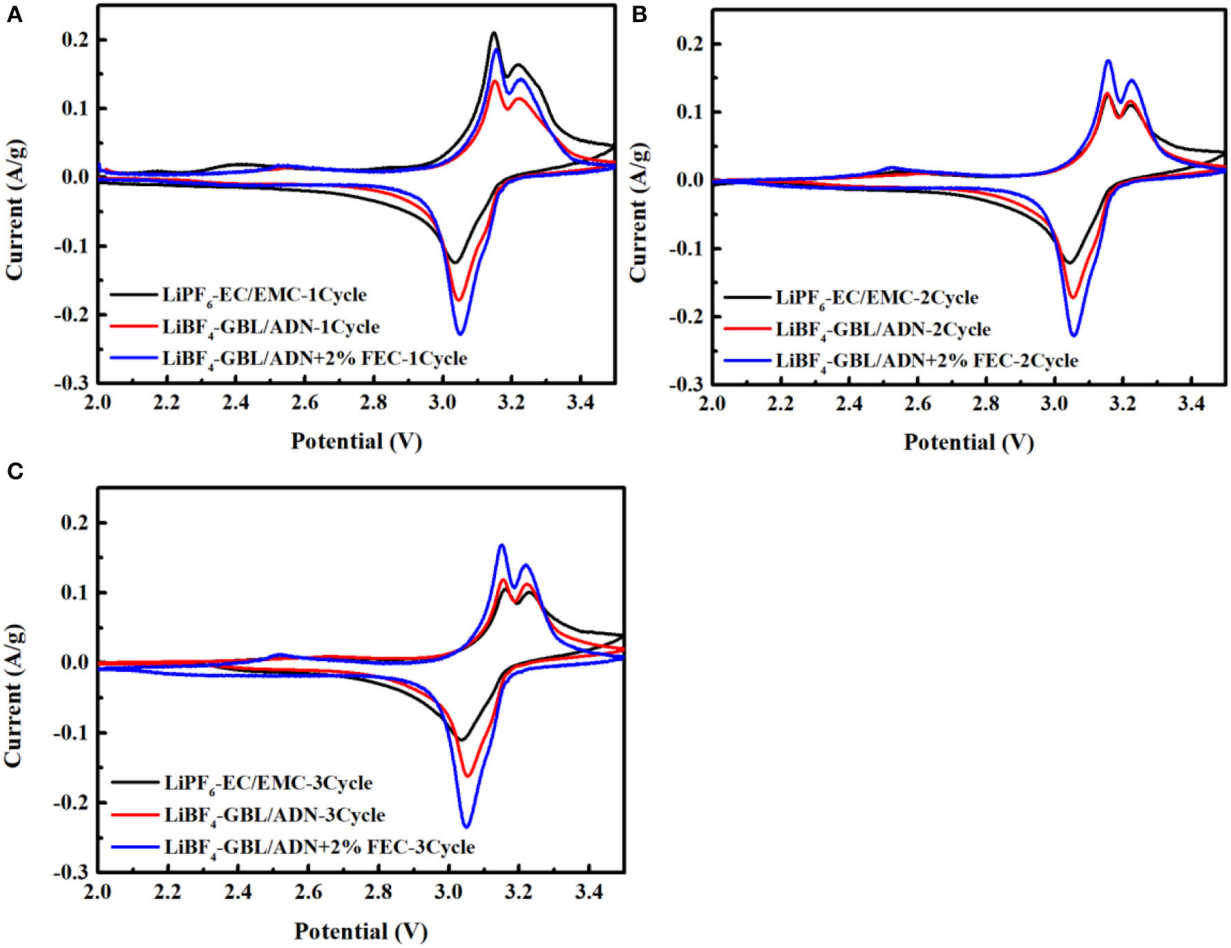
CV curve of the first three cycles of three electrolytes for LiNi_0.5_Mn_1.5_O_4_/ Li_4_Ti_5_O_12_ full-cells: **(A)** the first cycle; **(B)** the second cycle; **(C)** the third cycle.

Figure 1 shows the cyclic voltammetry test of the LNMO/LTO full battery in three electrolyte systems at room temperature, and the test conditions are 2–3.5 V, 0.01 mV s^−1^. The redox peak around 2.45 V in the figure belongs to Mn^3+^/Mn^4+^, which is caused by the oxidation reaction and the reduction reaction of Mn^3+^/Mn^4+^, when the LiNi_0.5_Mn_1.5_O_4_ active material is charged at a lower voltage. A small charge and discharge platform around 2.45 V can be corroborated. Two oxidation peaks appeared near 3.15 V, corresponding to Ni^2+^/Ni^3+^, Ni^3+^/Ni^4+^, but only one obvious reduction peak (Ni^3+^/Ni^2+^) and one weak reduction peak (Ni^4+^/Ni^3+^). This is because the oxidation state of Ni^3+^/Ni^4+^ is lower than the average oxidation state of the transition. Then in the process of Ni^3+^/Ni^4+^ transition, Li^+^ migration will encounter more repulsion. and in transition balance, Ni^3+^/Ni^4+^ is more balanced than Ni^2+^/Ni^3+^ transition, overcoming more energy barriers shows a very weak redox peak.

After the battery undergoes the first cycle, the CV peaks of the second and third cycles become well-matched and tend to be stable. In comparison, the CV peaks of the initial three cycles for the battery using the reference electrolyte exhibit the large changes of current, indicating that the SEI film formed during the oxidative decomposition of the reference electrolyte is unstable, causing the electrolyte to continue to decompose, and resulting in capacity loss. For the battery with FEC, the change of current is small, and after the third cycle, the peaks position difference between the oxidation peak and the reduction peak decreases from 0.1021 to 0.0933 in the first cycle. For the battery without FEC and the reference electrolyte, the peaks position difference decreases from 0.1090 to 0.0988 and 0.1211 to 0.1281, respectively, which shows that the reference electrolyte increases the polarization of the battery, and the LiNi_0.5_Mn_1.5_O_4_ electrode has a better intercalation and deintercalation performance in the electrolyte containing additives reversibility. It can be seen that the higher coulomb efficiency and rate performance was displayed during the charge and discharge process for the batter with FEC. In addition, from the second cycle, the CV curve area referring to the electrolyte with FEC is much larger than that to the reference electrolyte and the electrolyte without FEC. These phenomena show that the addition of FEC can provide a higher reversible capacity, which is consistent with the above results.

**Figure 2:**
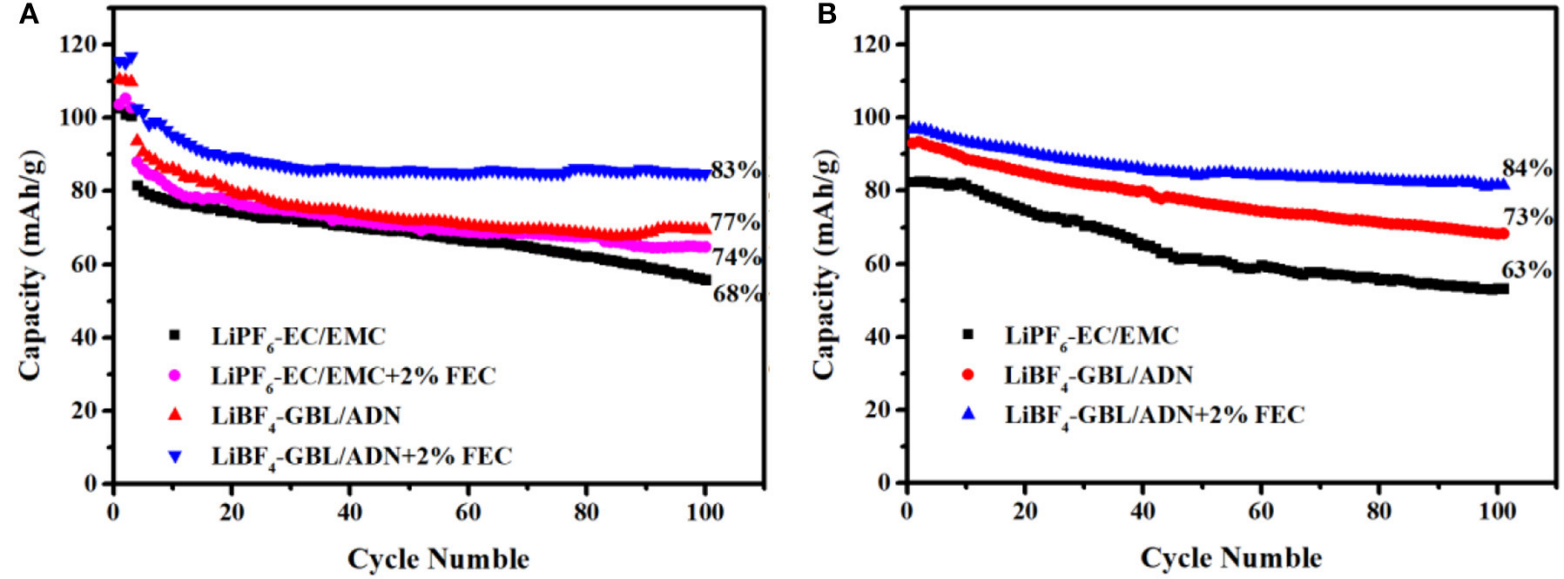
1C charge-discharge cycle performance in different electrolytes: **(A)** LNMO half-cells; **(B)** LNMO/LTO full-cells.

As shown in Figure 2A, the LNMO half-cell is charged and discharged at room temperature in different electrolytes. To ensure the full performance of the battery, the battery is charged and discharged three cycles at a current intensity of 0.1 C to achieve the purpose of activating the battery active material, and then cycle performance was obtained at 1 C. The purpose of adding FEC to the traditional electrolyte is used as a control group to judge the effect of FEC on the performance of the half-cell. It can be seen from the Figure 2A that in the initial three cycles of pre-circulation, the reference electrolyte and the comparison electrolyte 1st have a first-cycle discharge specific capacity of 102.7, 103.6 mAh g^−1^. After at 100 cycles, the capacity retention rate of the comparison electrolyte 1st (74%) is slightly higher than the reference electrolyte (68%), which means that adding FEC to the reference electrolyte is beneficial to stabilize electrode/electrolyte interface. Similarly, the first-cycle discharge specific capacity with the comparison electrolyte 2nd and the target electrolyte are 110.5 and 115.6 mAh g^−1^, respectively. After at 100 cycles, the capacity retention rate of the target electrolyte (83%) is higher than the comparison electrolyte 2nd (77%), which shows that target electrolyte has better electrochemical performance. It could be attributed to the positive electrode SEI film formed by FEC successfully restricting the direct contact between the electrode material and the electrolyte. The positive electrode SEI film could inhibit the decomposition of the electrolyte and the destruction of the electrode structure. Meanwhile, it is worth noting that the discharge specific capacity and capacity retention rate of the comparison electrolyte 2nd are higher than the reference electrolyte and the comparison electrolyte 1st. It is reason that ADN has a higher oxidation potential, it is not easy to be oxidized compared to carbonate solvents, and a higher proportion of ADN in the electrolyte solution preferentially occupies the active sites on the surface of the positive electrode material to hinder more carbonate solvents break down (Zhou et al., 2016). FEC combined with ADN could form a protective film with stable interface, and effectively improve the cycle performance of the battery.

This rule can be found in the full battery, that the Figure 2B is the cycle performance diagram of the LNMO/LTO full battery at room temperature for 100 cycles. The capacity retention rate and the specific discharge capacity of the target electrolyte (84%, 96.9 mAh g^−1^), and the comparison electrolyte 2nd (73%, 93 mAh g^−1^) are much higher than the reference electrolyte (63%, 82.5 mAh g^−1^). Whether it is the half-cell or the full-cell, because of the synergy of ADN and FEC, the battery shows excellent cycle performance.

With the widespread use of lithium-ion batteries, it is hoped that the battery can be fully charged in a shorter period. Therefore, higher requirements are placed on the power density of lithium-ion batteries, and the rate performance of the battery is the most important factor affecting its power density. Therefore, the development of an electrolyte with a good rate performance, is crucial to the further development of lithium ion batteries. Select the above ADN-based electrolyte and add FEC, using commercial electrolyte as a reference to further study its rate performance.

**Figure 3:**
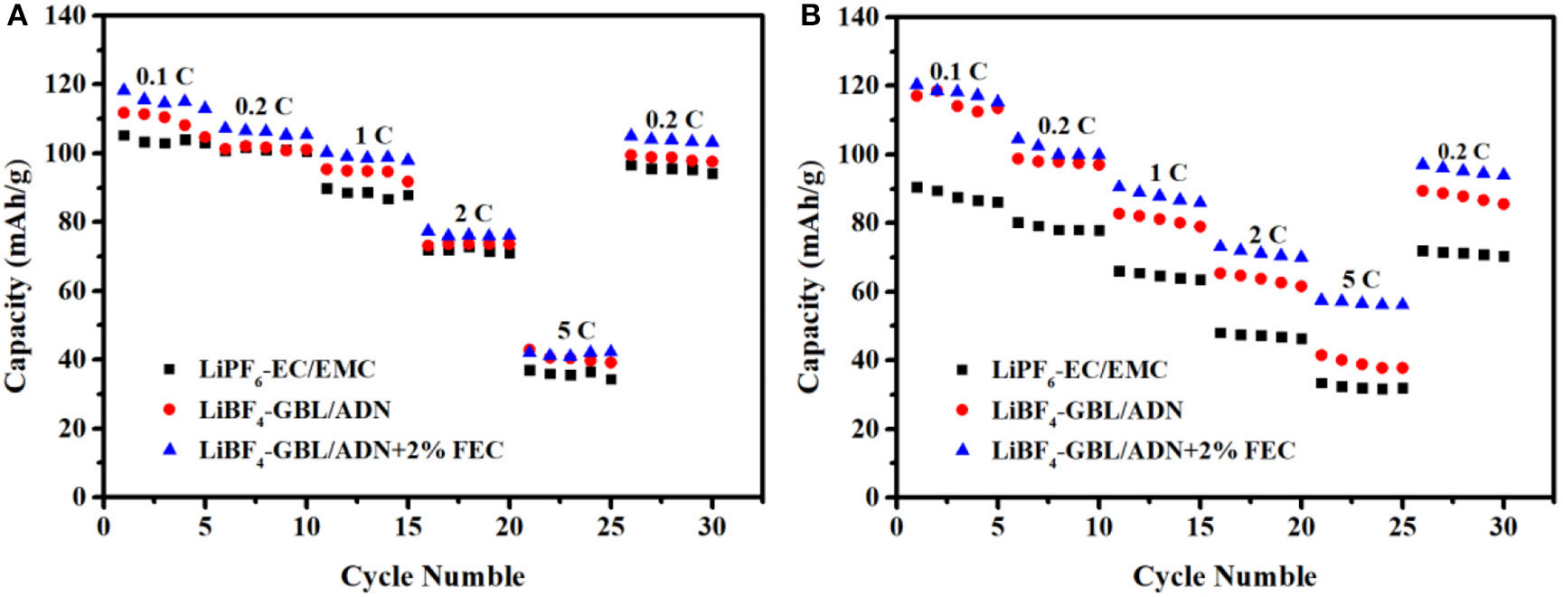
Rate performance in different electrolytes: **(A)** LNMO half-cells; **(B)** LNMO/LTO full-cells.

Figure 3A is the rate performance in different electrolytes of LNMO half-cells. When the current density increases, the discharge specific capacity gradually decreases. The main reason is that the heavily polarized at the large current density, and the polarization impedance increases rapidly. When the current density returns to 0.2 C, the discharge specific capacity also returns to the level of initial 0.2 C. Compared the target electrolyte with the comparison electrolyte 2nd shows the FEC additive not change the structure of the LiNi_0.5_Mn_1.5_O_4_ material after the battery undergoes rate cycling, and the electrode material is still intact.

The initial discharge specific capacity is 118.3, 111.8, 105.3 mAh g^−1^ for batteries with additives, no FEC and reference electrolytes, respectively. The target electrolyte shows more excellent rate performance, which is related to the synergistic effect of FEC and ADN. However, the effect of FEC is not obvious at the high current intensity, which shows that the migration rate of active substances in the electrolyte cannot keep up with the rate of electrochemical reactions at high current intensity. So that the electrolyte cannot provide enough free ions for the electrode surface, resulting in a lower capacity.

Figure 3B is rate performance in different electrolytes LNMO/LTO full-cell. It is worth noting that the addition of FEC in the ADN-based electrolyte makes the battery play a greater advantage. At 2 C current intensity, the specific discharge capacity of electrolytes with FEC, without FEC and reference electrolyte are 73.1, 65.4, and 48.1 mAh g^−1^, respectively, which are corresponding to 60.8, 55.8, and 53.1% at 0.1 C. Obviously, the adiponitrile synergistic fluoroethylene carbonate electrolyte has better compatibility for the full battery with Li_4_Ti_5_O_12_ as the negative electrode than the reference electrolyte at the high current intensity. It meets the higher requirement of power density application of lithium ion battery. The results showed that the full battery containing FEC electrolyte exhibits more excellent rate performance under high pressure, which is consistent with the results obtained from the half-cell research.

**Figure 4:**
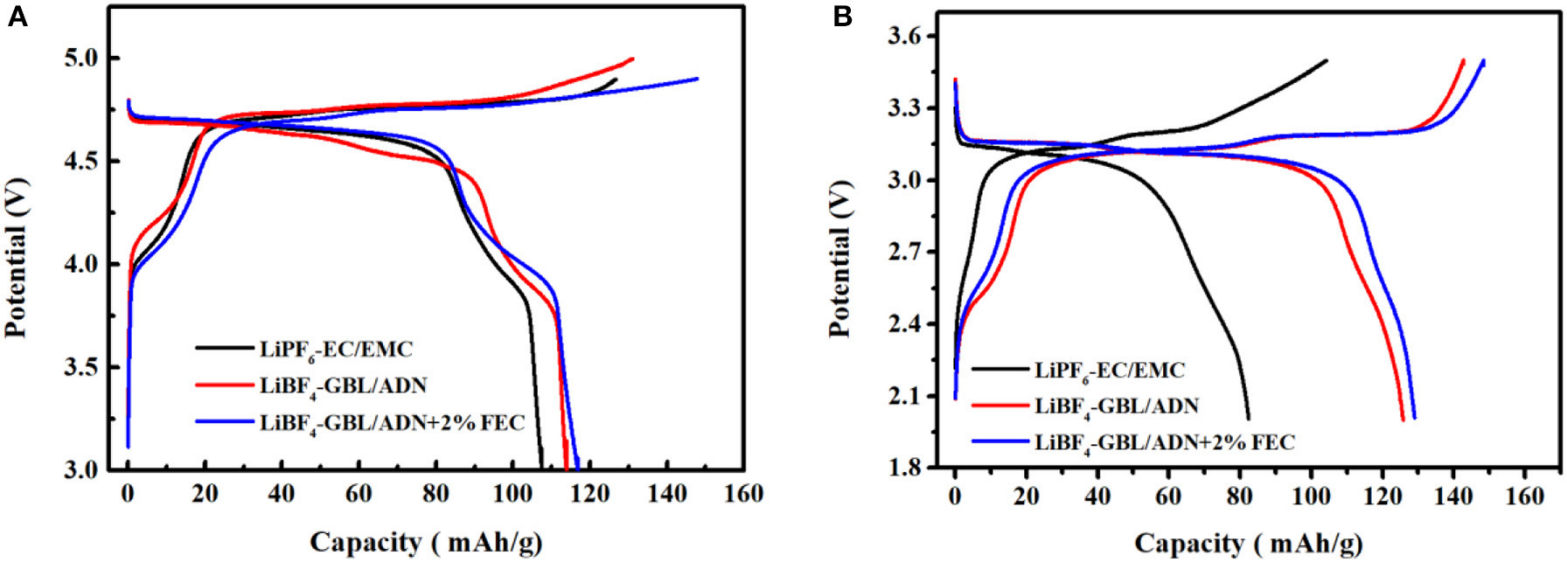
Charge and discharge curve at the first cycle of three electrolytes at 0.1 C rate: **(A)** LNMO half-cells; **(B)** LNMO/LTO full-cells.

Figure 4A is the first charge-discharge curve of LNMO/Li half-cell in the range of 3.0–4.9 V at 0.1 C current intensity. The specific discharge capacity of the battery with FEC electrolyte in the first cycle (116.8 mAh g^−1^) is better than that without FEC (113.9 mAh g^−1^) and the reference electrolyte (107.5 mAh g^−1^). It can be seen that the charge-discharge curve has two charge and discharge platforms of 4.0 and 4.7 V in three different electrolyte systems, the 4.0 V charge and discharge platform corresponds to the Mn^4+^/Mn^3+^ redox in the material. The 4.7 V charge and discharge platform belongs to the Ni^4+^/Ni^2+^ redox, and this platform is relatively stable. Compared with the reference electrolyte, the battery containing the FEC electrolyte has a higher coulomb efficiency (76.5%) of the first cycle battery, which indicates that the addition of FEC has better chemical reaction kinetic activity in batteries. The electrolyte oxidizes and decomposes to produce an initial protective film, the protective film repairs and grows self during the battery proceeds. Finally, with the battery charge and discharge process continues, the interface film tends to stabilize and the oxidative decomposition of electrolyte gradually suppressed, resulting in the columbic efficiency of the battery close to 100%.

Figure 4B is the first charge-discharge curve of LNMO/LTO full battery. There are two charge-discharge platforms. The reference electrolyte has a weak redox platform in the 2.4–2.7 V range. This indicates that the reference electrolyte has almost no chemical reaction with manganese ions, rarely participates in the generation of SEI film on this platform, and consumes less electrolyte, which also shows that the battery led by the reference electrolyte has a low impedance at the beginning of the cycle. However, at the second charge and discharge platform 3.14–3.21 V corresponding to the Ni^4+^/Ni^2+^ redox couple, the reference electrolyte battery has a very low discharge platform compared to the other two ADN-based electrolyte batteries. Meanwhile, the charging platform is high and long, and the distance between the charge and discharge curves is large. This shows that the polarization is very large, the compatibility between the reference electrolyte and the LNMO full-cells with LTO as the negative electrode is not good, resulting in a low specific discharge capacity and coulomb efficiency (82.4 mAh g^−1^ and 46.8%). For the ADN-based electrolyte battery, the distance between the charge and discharge curves is small and the platform is very stable, resulting in low polarization. Batteries with FEC electrolyte have better electrochemical stability, and the LNMO energy of FEC is relatively low, so reduction decomposition could occur at a higher electric potential, thus forming the SEI film with more stable composition and structure. Generally, better SEI film means the better cycling performance.

**Figure 5:**
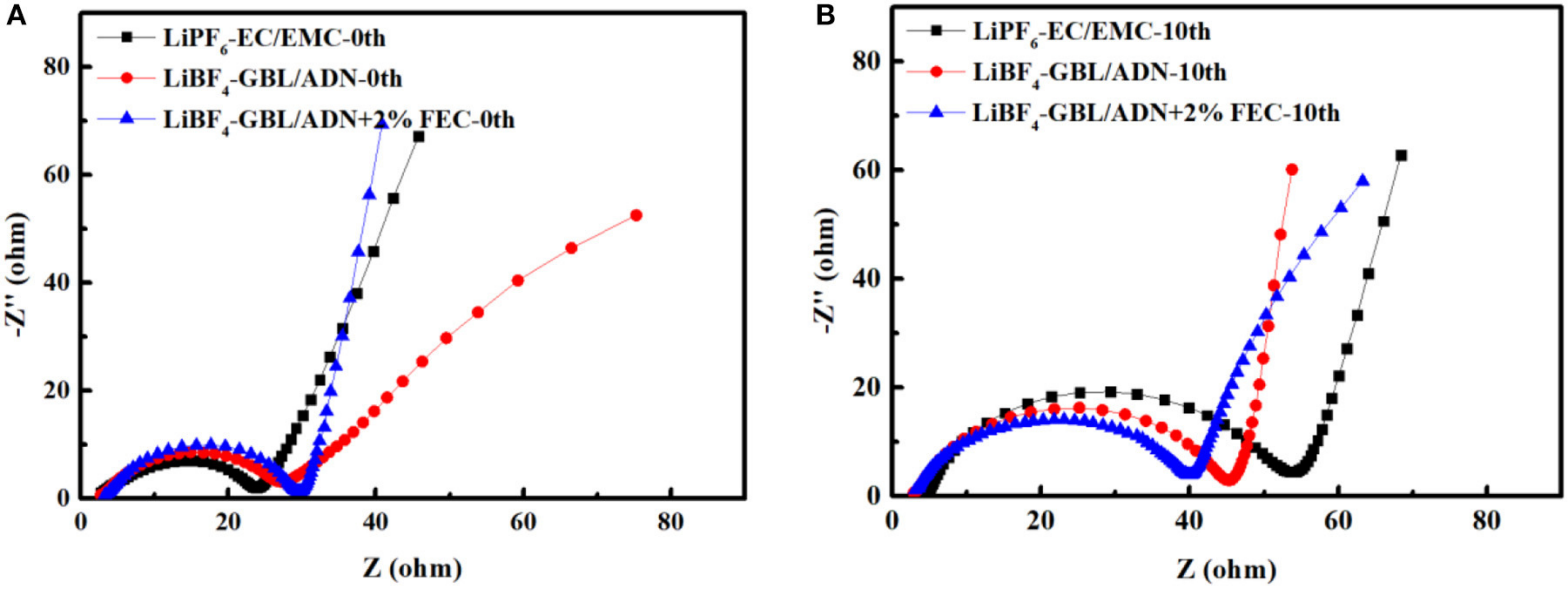
EIS spectrum of LNMO/Li battery before and after 0.1 C rate cycling: **(A)** before cycled; **(B)** after 10 cycles.

The EIS spectrum of a battery is usually described by four main characteristics. The high-frequency and intermediate-frequency flat semicircles can be attributed to the interface film (Rf) and lithium ion charge transfer (Rct) at the interface of the film and the active material (coupled to the thin film and/or interface capacitance). Low-frequency Warburg-type elements reflect the solid-state diffusion of lithium ions. Finally, at very low frequencies, the Z vs. Z″ curve becomes a steep, almost vertical line, which reflects the capacitive behavior of the electrode (lithium ion insertion and corresponding charge transfer) (Ou et al., 2012). Figure 5A is the AC impedance measured by the LNMO half-cell before cycling. It can be seen that the interfacial resistance (22.3 Ω) of the reference electrolyte battery is the smallest. It means that in the initial formation reaction, the reference electrolyte not participate or rarely participates in the generation of SEI film on this platform, so that consumes less electrolyte. The battery with FEC electrolyte has a maximum interface impedance (25.6 Ω), which shows that FEC directly participates in the formation of the interface film and consumes a large amount of electrolyte, making the charge and discharge efficiency lower in the first cycle. This corresponds to the corollary of Figure 4. The formation of the film has double effects. On the one hand, the formation of the film consumes lithium ions in the electrolyte or electrode. The thickness of the film increased could improve the polarization of the battery, and resulting in a decrease of rate performance in battery. On the other hand, the formation of the film improves the cycle performance of the battery, especially when the film was formed stable and relatively thin, thereby improving the overall performance of the battery (Ma et al., 2018). Generally, the SEI film initially formed by the oxidative decomposition of the electrolyte after 10 cycles. As shown in Figure 5B, the resistance of the reference electrolyte increased to 57.6 Ω, the comparative electrolyte 2nd and the target electrolyte increased to 43.1 and 40 Ω. It shows that FEC is more conducive to the formation of a stable SEI film, and inhibiting the continuous reaction between the electrolyte and the electrode consumes the electrolyte. Therefore, the lithium ions could to be effectively extracted and intercalated and improving the electrochemical performance of the battery.

**Figure 6:**
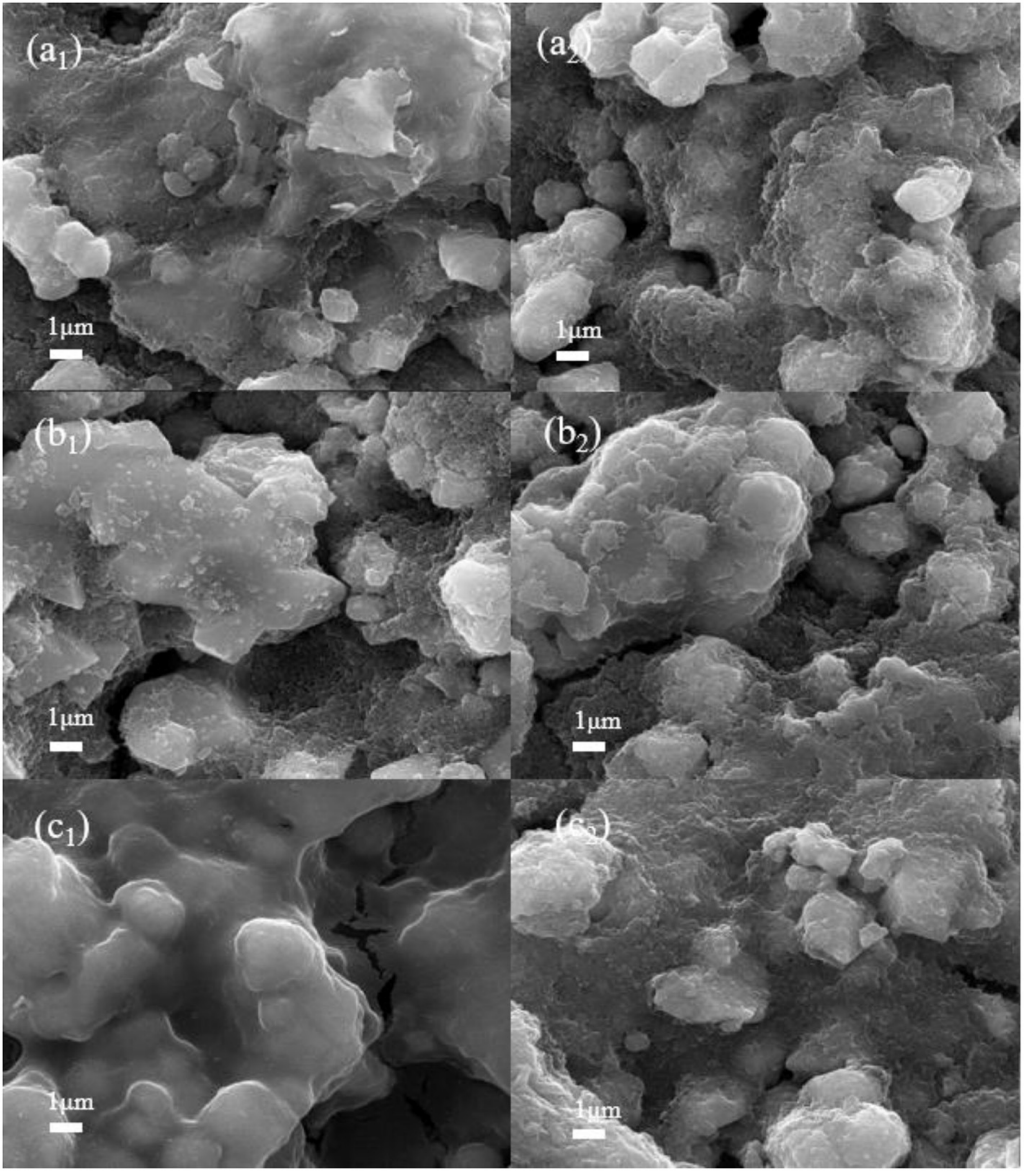
SEM images of LiNi_0.5_Mn_1.5_O_4_ cathode surface of three electrolytes after 10 and 100 cycles: **(a**_**1**_**,a**_**2**_**)** 1 mol L^−1^ LiPF_6_-EC/EMC; **(b**_**1**_**,b**_**2**_**)** 2 mol L^−1^ LiBF_4_-GBL/ADN; **(c**_**1**_**,c**_**2**_**)** 2 mol L^−1^ LiBF_4_-GBL/ADN+2%FEC.

In order to further explore the formation mechanism of the electrolyte interface film at the electrode/electrolyte interface, the LiNi_0.5_Mn_1.5_O_4_ batteries of the commercial electrolyte, the target electrolyte and the comparative electrolyte 2nd were disassembled after 10 and 100 cycles, respectively. The pole pieces were cleaned by the AN several times, and then transferred to the vacuum drying oven at 120°C for 2 h. The uniformity of the interface film on the surface of the material and the changes in the structure are characterized by SEM. Figures 6a_1_,a_2_ are SEM images of 1.0 M LiPF_6_-EC/EMC cathode surface after 10 and 100 cycles, respectively. It can be seen that the surface of the electrode is rough and fuzzy is due to the surface of the LiNi_0.5_Mn_1.5_O_4_ particles covered with electrolyte decomposition products, which polymerize into the film and adhere to the surface of the material unevenly. With the progress of reaction, the surface morphology of the material after the 100th was deeper than that of the 10th, and it shows that a slight collapse of the structure of LiNi_0.5_Mn_1.5_O_4_ material has a slight collapse and cracks in the particles (Ma et al., 2018). It could be the commercial electrolyte reacts the continuous oxidative decomposition reaction at 4.9 V, and producing strong corrosive HF, which causes its different valences of nickel and manganese ions to dissolve in the electrolyte (Ou et al., 2012). As a result, the structure of the material was destroyed and the charge and discharge performance of the battery was reduced seriously. Similarly, in Figures 6b_1_,b_2_ that the surface of the battery pole piece after the cycle of the comparative electrolyte 2 not form a good morphology, the surface is relatively rough, and the formed interface film is not obvious. It could be because of the ADN not exert the advantage of high-voltage at the 4.9 V LNMO. However, its surface is better than the battery of the reference electrolyte.

In contrast, Figure 6c_1_ shows the morphology of LiNi_0.5_Mn_1.5_O_4_ after 10 cycles in 2 mol L^−1^ LiBF_4_-GBL/ADN+2% FEC electrolyte. It can be clearly observed that a dense and uniform interface film is attached to the surface of the material, and it is flat and smooth. This interface film cane effectively prevents electron transport and solvent molecule shuttle, but provides a channel for the migration of lithium ions during the cycle, effectively isolating the electrolyte from contact with LiNi_0.5_Mn_1.5_O_4_ active particles, which further inhibits the decomposition of electrolyte and provide a good protection for the positive electrode. However, as shown in Figure 6c_2_, when the battery is cycled to 100 cycles, the smoothness of the interface film is impaired, which leads to an increase in battery impedance and affects a decrease in discharge capacity. This trend is consistent in ADN-based batteries without FEC additives. In general, FEC electrolyte additives can promote the formation of a stable interface between electrode and electrolyte under the effect of ADN high pressure resistance, which is the reason for the FEC can improve battery performance as an electrolyte additive.

**Figure 7:**
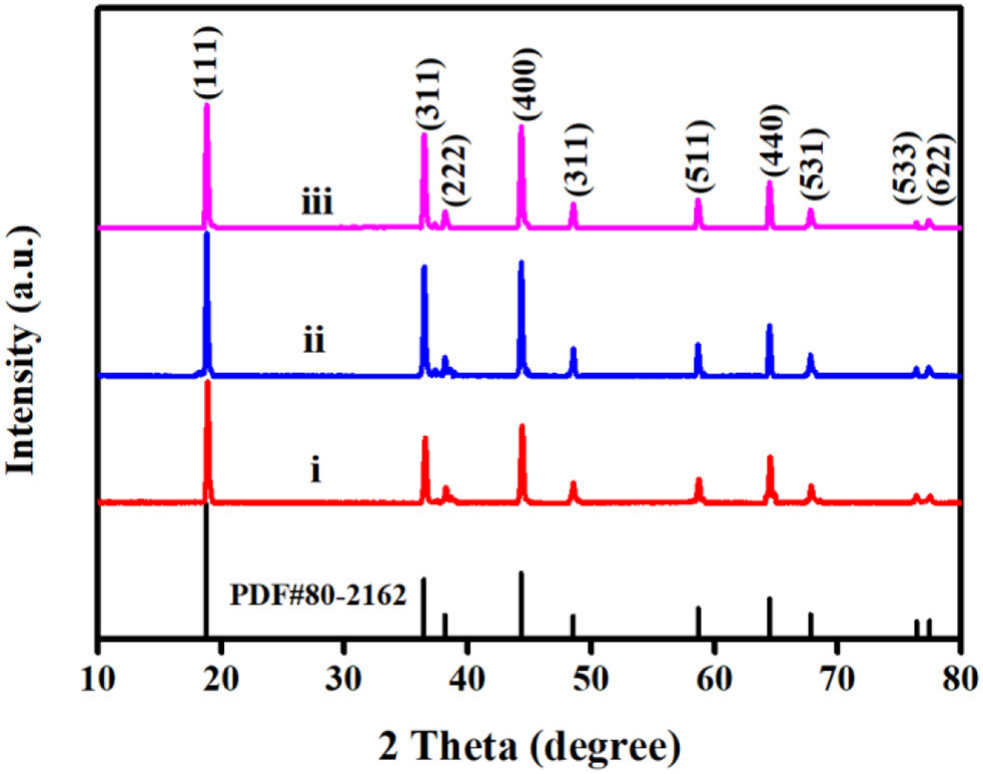
XRD patterns of LiNi_0.5_Mn_1.5_O_4_ after 20 cycles with three electrolytes: (i) 1 mol L^−1^ LiPF_6_-EC/EMC; (ii) 2 mol L^−1^ LiBF_4_-GBL/ADN; (iii) 2 moL^−1^ LiBF_4_-GBL/ADN+2%FEC.

Figure 7 investigates the X-ray diffraction (XRD) pattern of LiNi_0.5_Mn_1.5_O_4_ after 20 cycles of three electrolytes. The diffraction peaks of the three samples are well-matched with the standard PDF#80-2162 card of LiNi_0.5_Mn_1.5_O_4_ material, it shows that the pole piece material has not changed original spinel structure after the three electrolyte cycles. For the commercial electrolyte battery, the (111) diffraction peak of the material after cycling is relatively lowest, which indicates the commercial electrolyte cannot prevent the crystallinity of the material from decreasing at the high pressure of 4.9 V. Because the lower crystallinity can affect the electrochemical performance of materials. Compared with the comparative electrolyte 2 battery, the pole piece of target electrolyte battery has a stronger intensity of the (111) diffraction peak, with the narrow half-width and sharp peak shape, which proves that its crystallinity is relatively good (Liu et al., 2015). According to reports in the literature, the ratio of the (311) diffraction peak to the (111) diffraction peak can determine the degree of material cation mixing. When the ratio is smaller, the structure of the material is more stable in a disordered state (Wu et al., 2017). Based on calculation, the I (311)/I (111) values of commercial electrolyte batteries, target electrolyte batteries and comparative electrolyte are 0.766, 0.753, and 0.724, respectively. It is indicating that the degree of cation mixing of batteries with FEC additives is lower, and the crystal structure is more stable.

**Figure 8:**
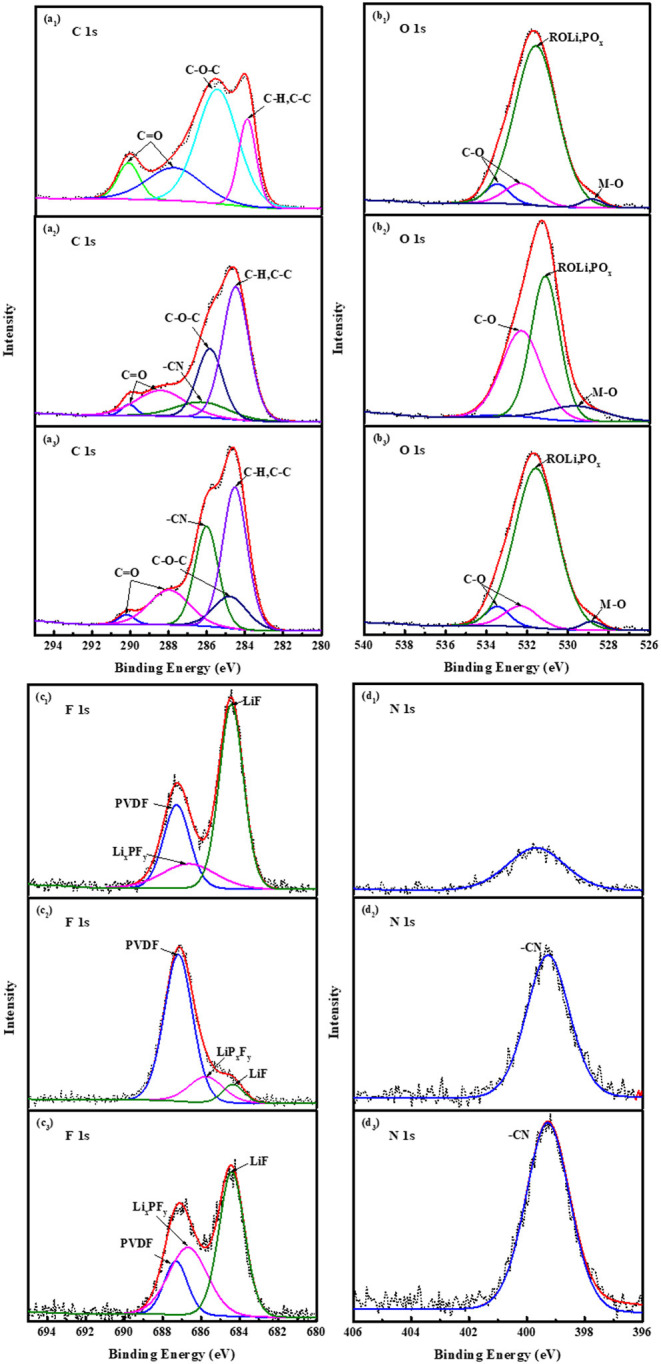
XPS spectra of LiNi_0.5_Mn_1.5_O_4_ cathodes of C 1s, O 1s, F 1s and N 1s with three electrolyte after 20 cycles: **(a**_**1**_**-d**_**1**_**)** 1 mol L^−1^ LiPF_6_-EC/EMC; **(a**_**2**_**-d**_**2**_**)** 2 mol L^−1^ LiBF_4_-GBL/ADN; **(a**_**3**_**-d**_**3**_**)** 2 mol L^−1^ LiBF_4_-GBL/ADN+2%FEC.

Figure 8 is X-ray photoelectron spectroscopy (XPS) spectra of LiNi0.5Mn1.5O4 cathodes of C 1s, O 1s, F 1s, and N1s with three electrolytes after 20 cycles. In the C 1s spectrum of the three electrolyte batteries, the peak at 285.4 eV is attributed to alkyl lithium carbonate (C-O-C), and the peak at 290.8 and 287.8 eV belong to inorganic lithium carbonate (C=O). During the cycle, the generation of peaks indicates that there are inorganic substances such as Li_2_CO_3_ and organic substances ROCO_2_Li and ROLi on the surface of the positive electrode material, which mainly come from the decomposition products of lithium carbonate, lithium carbonate electrolyte and trace water (Rong et al., 2014; Zhang et al., 2016). The existence of these substances can be further proved in the O 1s spectrum, the C-O peak is at 534.2 eV, and the C=O peak is at 532.8 eV (Eriksson et al., 2002). It is worth noting that the C-O-C on the surface of the electrode with FEC is weak, and the C=O peak is strong, which shows that FEC effectively inhibits the oxidative decomposition of the electrolyte. It is the same as the tendency of the C-O peak in the O 1s spectrum to be weak. The peak at 284.5 eV in the C 1s spectrum is attributed to the C-C bond in the hydrocarbon (C-H) and conductive carbon black (An et al., 2011; Ha et al., 2013). In addition, in the C 1s spectrum of the battery material containing ADN groups, as shown in Figures 8a_2_,a_3_, the presence of the -CN group was detected at 286.5 eV, which also corresponds to 399.5 eV in the N 1s spectrum (Deniau et al., 1992). At the high-pressure conditions, it fully indicates that ADN molecules directly participate in the formation of the positive electrode interface film. The -CN group is the main component of the SEI film, which ensures the migration of lithium ions between the positive and negative electrodes, and it is beneficial to improve the cycling stability of the electrode.

As shown in Figure 8c_1_, the characteristic peak at 687.9 eV belongs to the C-F bond in PVDF, and the strong peak at 684.5 eV belongs to LiF in the F_1s_ spectrum. Inorganic LiF mainly comes from the thermal decomposition reaction of lithium salt LiPF_6_ and trace water in the electrolyte (Han et al., 2010; Li et al., 2013). It will be accompanied by the formation of high-strength corrosive acid (HF). The corrosive acid will initiate a series of autocatalytic decomposition reactions involving the electrolyte and the transition metal oxide cathode, and severely destroying the formation of the interface film. Compared with the other two ADN-based electrolyte electrodes, the LiF peak containing LiPF_6_ is relatively strong, resulting in a strong charge impedance, indicating the reason why the traditional electrolyte has poor cycling stability for LiNi_0.5_Mn_1.5_O_4_ material at the high voltage of 4.9 V. As shown in Figures 8c_2_,c_3_, it also corresponds to LiF at 684.5 eV, which is the self-decomposition reaction from LiBF_4_. For the LiF peak containing FEC electrolyte batteries, a small part is the self-decomposition reaction of LiBF_4_, and more is the fluorine group in the fluoroethylene carbonate. The group has a strong electron-withdrawing ability. At higher reduction potentials, fluorine-containing substances such as Li_x_PF_y_ and LiF (Michan et al., 2016) generated by the reaction preferentially occupy the active site of LiNi_0.5_Mn_1.5_O_4_, meanwhile, it suppress low reduction potential electrolyte solvents break down. The XPS results further show that the ADN-based electrolyte containing FEC additives makes the positive electrode LiNi_0.5_Mn_1.5_O_4_ beneficial to the formation of the SEI film, and improves the cycle stability and rate performance.

## Conclusions

The electrochemical performance referring to 5 V-level LiNi_0.5_Mn_1.5_O_4_ cathode material was studied in the high voltage electrolyte system (2 mol L^−1^ LiBF_4_-GBL/ADN+2% FEC). Compared with the traditional commercial electrolyte and FEC-free electrolyte systems, the FEC system can improve the battery cycle stability. At 1 C rate, the 100-cycled capacity retention rate can reach 83%, while the capacity retention rate without FEC system and commercial electrolyte system is only 77% and 68%, and the rate performance has been improved. The results of EIS and CV curves for LiNi_0.5_Mn_1.5_O_4_ batteries show that the synergistic effect of ADN and FEC can reduce their polarization at high voltages. The interface film between the electrolyte and the electrode tends to be stable, thus reducing capacity loss and improving battery electrochemical performance. Fluorine can preferentially occupy the active site of LiNi_0.5_Mn_1.5_O_4_ and inhibit the decomposition of the electrolyte with low reduction potential, finally stabilizing the electrochemical performance of the electrode. The electrochemical performance of LiNi_0.5_Mn_1.5_O_4_/Li_4_Ti_5_O_12_ in the high-voltage electrolyte mentioned above shows that FEC is a good film-forming additive with a wide ADN window at high voltage. It has a good compatibility for all battery materials capacitive and plays an important role in promoting the application of high-voltage batteries.

## Data Availability Statement

All datasets presented in this study are included in the article/supplementary material.

## Author Contributions

ZF, ZZ, and WC proposed the idea. ZZ, WC, and XZ prepared all materials. ZZ, WC, and KZ conducted electrochemical experiments and analyzed the data. ZF, ZZ, WC, and XZ wrote the manuscript. ZF, WC, and LL supervised the implementation of the study. All authors contributed to this article and approved the submitted version.

## Conflict of Interest

The authors declare that the research was conducted in the absence of any commercial or financial relationships that could be construed as a potential conflict of interest.
